# Relationship between brain iron deposition and mitochondrial dysfunction in idiopathic Parkinson’s disease

**DOI:** 10.1186/s10020-021-00426-9

**Published:** 2022-03-04

**Authors:** Jannik Prasuhn, Martin Göttlich, Friederike Gerkan, Sofia Kourou, Britt Ebeling, Meike Kasten, Henrike Hanssen, Christine Klein, Norbert Brüggemann

**Affiliations:** 1 Institute of Neurogenetics, University of Lübeck, Ratzeburger Allee 160, 23538 Lübeck, Germany; 2 Department of Neurology, University Medical Center Schleswig-Holstein, Campus Lübeck, Lübeck, Germany; 3 Center for Brain, Behavior, and Metabolism, University of Lübeck, Lübeck, Germany; 4 Department of Psychiatry and Psychotherapy, University Medical Center Schleswig-Holstein, Campus Lübeck, Lübeck, Germany

**Keywords:** Parkinson's disease (PD), Mitochondria, Iron

## Abstract

**Background:**

The underlying pathophysiology of Parkinson's disease is complex, involving different molecular pathways, including brain iron deposition and mitochondrial dysfunction. At a molecular level, these disease mechanisms are likely interconnected. Therefore, they offer potential strategies for disease-modifying treatments. We aimed to investigate subcortical brain iron deposition as a potential predictor of the bioenergetic status in patients with idiopathic Parkinson’s disease.

**Methods:**

Thirty patients with idiopathic Parkinson's disease underwent multimodal MR imaging (T1, susceptibility-weighted imaging, SWI) and ^31^phosphorus magnetic resonance spectroscopy imaging. SWI contrast-to-noise ratios served as a measure for brain iron deposition in the putamen, caudate, globus pallidus, and thalamus and were used in a multiple linear regression model to predict in-vivo energy metabolite ratios.

**Results:**

Subcortical brain iron deposition, particularly in the putamen and globus pallidus, was highly predictive of the region-specific amount of high-energy-containing phosphorus metabolites in our subjects.

**Conclusions:**

Our study suggests that brain iron deposition but not the variability of individual volumetric measurements are highly predictive of mitochondrial impairment in vivo. These findings offer the opportunity, e.g., by using chelating therapies, to improve mitochondrial bioenergetics in patients with idiopathic Parkinson's disease.

**Supplementary Information:**

The online version contains supplementary material available at 10.1186/s10020-021-00426-9.

## Background

Various molecular disease mechanisms are associated with nigral and extranigral neurodegeneration in patients with Parkinson's disease (PD), often determining disease onset and progression (Timpka et al. 2017). Two such molecular alterations involve brain iron homeostasis and mitochondrial function disturbances (Muñoz et al. 2016). Although the concept of mitochondrial dysfunction involves distinct pathophysiological aspects, e.g. impaired mitophagy and altered mitochondrial dynamics, the final common pathway is bioenergetic depletion (Park et al. 2018). The underlying idea that iron metabolism changes and mitochondrial disturbances are relevant for the disease development refers to the initial, environmental agent-related studies (involving MPTP, 6-OH-DOPA, rotenone, or paraquat), that impair mitochondrial homeostasis (Gaki and Papavassiliou 2014). In-vivo models have furthered our understanding of these environmental agents, revealing increased iron deposition in subcortical brain structures following mitochondrial impairment (Mochizuki et al. 1994). Subcortical brain iron deposition in PD has been extensively studied using neuroimaging and post-mortem brain examinations (Zhang et al. 2010; Trufanov et al. 2019; Barbosa et al. 2015; Wang et al. 2016). Histopathological investigations have demonstrated that iron deposition is mainly localized to the mitochondria on a subcellular level, stressing the importance of this organelle in regulating intracellular iron metabolism (Muñoz et al. 2016). Mitochondria are responsible for the macromolecular iron integration in metalloproteins, Fe-Sulfur clusters, or heme groups (Liang and Patel 2004; Mena et al. 2011). Previous reports highlighted that increased oxidative stress (e.g., by the inhibition of complex I of the electron transport chain) leads to an impaired assembly of Fe-Sulfur clusters, forcing the mitochondria to import even more iron (Lee et al. 2009). This action might be a self-promoting mechanism as the resulting disproportion of divalent and trivalent iron could increase oxidative stress (Moos and Morgan 2004). Based on these in vitro interactions, the aggravating effects of iron and mitochondrial dyshomeostasis would be reasonable to study for the potential development of disease-modifying treatment strategies (Kakhlon et al. 2010; Sohn et al. 2008; Devos et al. 2020). In this context, specific chelating agents cross the blood–brain barrier, e.g. deferoxamine or deferiprone, might thus rescue iron-overloaded mitochondria by cellular iron redistribution (Kakhlon et al. 2010; Chan et al. 2018). To the best of our knowledge, non-invasive studies combining brain iron deposition (by susceptibility-weighted imaging, SWI) and bioenergetic depletion (by ^31^phosphorus magnetic resonance spectroscopy imaging, ^31^P-MRSI) have not yet been performed in PD. Therefore, our primary hypothesis was to test whether (i) subcortical brain iron deposition or (ii) the individual volumetric measurements are predictive of bioenergetic depletion in patients with PD. The combination of these two imaging modalities might not only help to recapitulate *in-vitro* and preclinical *in-vivo* findings to understand disease pathophysiology in human subjects but might also serve as a measure of patient stratification in future clinical trials.

## Methods

### Recruitment and clinical assessment

The present study and all subsequent experimental procedures have been performed in accordance with the revised version of the Declaration of Helsinki. Before the enrollment of the first study participant, this study has been approved by the ethics committee of the University of Lübeck (AZ 18_945). All study participants gave written informed consent before participation in this study. We confirmed the diagnosis of PD following the MDS clinical diagnostic criteria as evaluated by trained movement disorders specialists (JP, HH, NB) (Postuma et al. 2015). The clinical examination included the general patient history and demographic data, potential MRI contraindications, concomitant illnesses, medication including the levodopa-equivalent daily dosage (LEDD), and a standardized clinical assessment following the MDS-UPDRS protocol (Goetz et al. 2008). All patients were regularly taking antiparkinsonian medication, were in the ON state, not fasting, and rested for at least one hour before the start of the imaging procedure.

### MRI sequences and analyses

All MRI measurements were performed at the CBBM Core Facility Magnetic Resonance Imaging on a 3 T Siemens MAGNETOM Skyra Magnetic Resonance Imaging scanner.

#### T1 imaging

We employed a three-dimensional T1-weighted MP-RAGE sequence (64-channel head/neck coil) for structural imaging following subsequent imaging parameters: 1 × 1 × 1 mm^3^ voxel size; 192 × 256 × 256 mm^3^ field of view; TR = 1900 ms; TE = 2.44 ms; TI = 900 ms; flip angle 9°; GRAPPA acceleration factor 2 along anterior/posterior phase-encoding direction, total scan time 4 min and 33 s). All acquired neuroanatomical images were evaluated by consulting neuroradiologists to preclude relevant brain lesions.

#### Susceptibility-weighted imaging

We performed SWI using a standard Siemens 3D high-spatial-resolution fully velocity corrected gradient-echo MRI sequence with the following image parameters: voxel size 0.9 × 0.9 × 1.5 mm^3^; 220 × 220 × 120 mm^3^ field of view; TR = 27 ms; TE = 20 ms; flip angle 15°; transversal orientation; right/left phase-encoding direction; total scan time 4 min and 54 s).

#### ^31^Phosphorus magnetic resonance spectroscopy imaging

**Fig. 1:**
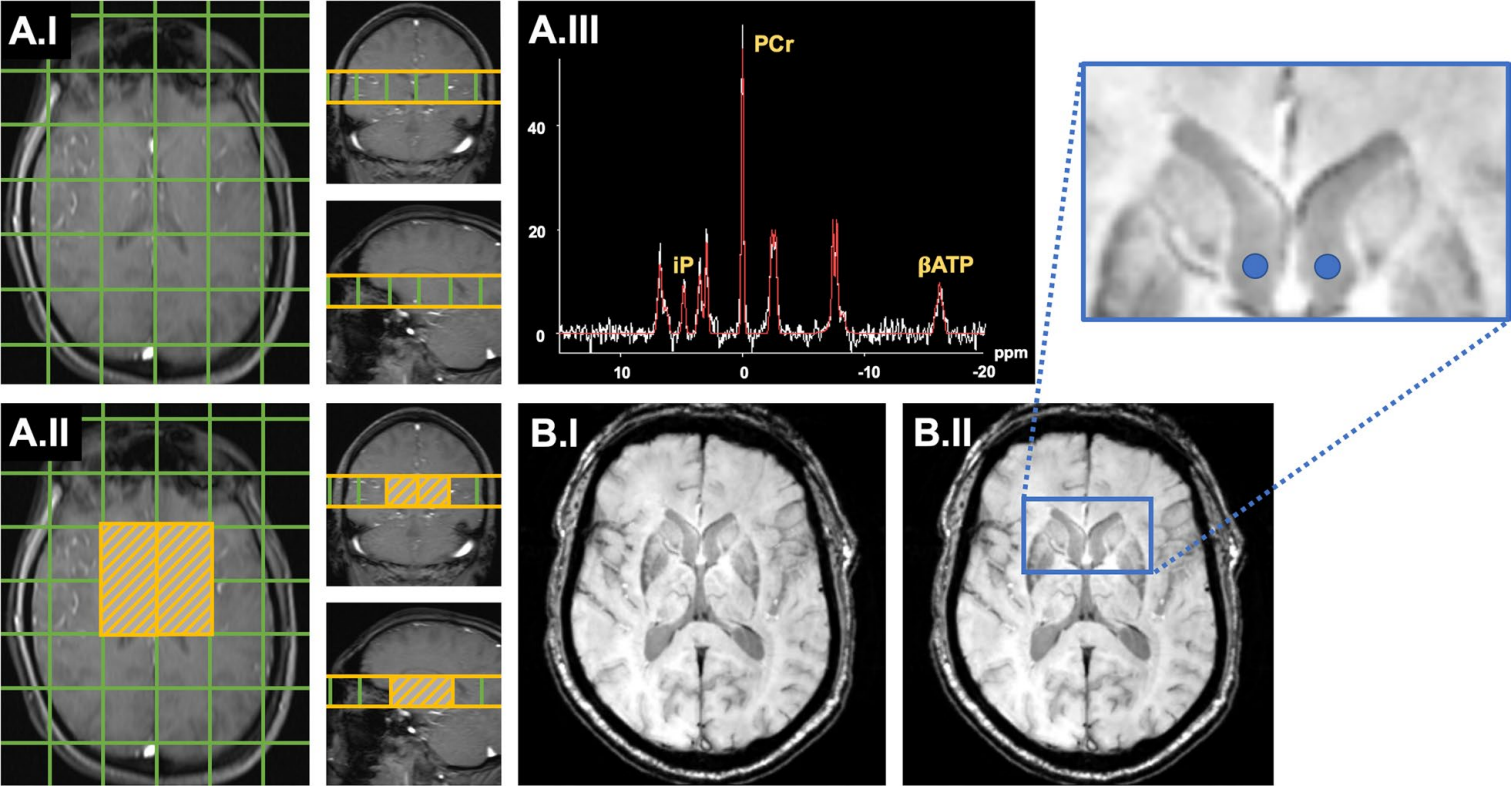
Methodological approaches for the analysis of the multimodal neuroimaging data. In **A**, analyses of ^31^P-MRSI measurements are summarized; in **B**, the approach on calculations of CNRs (as derived from SWI). Panel A.1 illustrates the voxel size and CSI grid placement (green) for ^31^P-MRSI measurements in axial, coronal, and sagittal planes. In Panel A.II, the voxels of interest (VOIs) for subcortical brain regions are highlighted for each hemisphere (orange hatched). One exemplary ^31^P-MRSI spectrum (white line) and the respective model line fit (red line) is shown in Panel A.III. The metabolites of relevance for this study are labeled in yellow. For the sake of readability, other peaks are not marked, as they were not of interest to the hypothesis of this study. In Panel B.I, an exemplary SWI image of one study participant in the axial plane is shown. In Panel B.II, we highlighted the reference ROI placement (blue circles) in the lateral ventricles by a magnified snippet (blue framework). *31P-MRSI* ^31^Phosphorus magnetic resonance spectroscopy imaging. *ATP* adenosine triphosphate, *CSI* chemical shift imaging, *iP* inorganic phosphate, *PCr* phosphocreatinine, *ppm* parts per million, *ROI* region of interest, *SWI* susceptibility-weighted imaging, *VOI* voxel of interest

We used a dual tuned quadrature head coil ^1^H/^31^P (RAPID Biomedical) for 3 T and applied a 3D Chemical Shift Imaging (CSI) Free Induction Decay sequence (CSI-FID) to acquire MRSI data. The protocol parameters were as follows: voxel size 30 × 30 × 30 mm^3^; 240 × 240 × 240 mm^3^ field of view; TR = 2000 ms; TE = 2.3 ms; sixfold weighted averaging; flip angle 50°; spectral bandwidth 2000 Hz; vector size 1024; Hamming filtering (width 100%); Nuclear overhauser effect disabled; WALTZ-4 decoupling, total scan time 8 min and 4 s. CSI grid placement and the volume of interest covering the basal ganglia are highlighted in Fig. 1. Adjustments and shimming were performed on a manually selected volume that was slightly larger than the volume of interest.

#### Neuroimaging analyses

##### Volumetric analysis

We performed volumetric measurements of subcortical brain structures on T1 native space images following the segmentation via the well-established FIRST suite of the FMRIB software library (v6.0) (Jenkinson 2008; Patenaude et al. 2011). We have chosen the respective options to derive only the left- and right-sided segmentation of the putamen, caudate, globus pallidus, and thalamus. Images of all subjects were manually controlled for segmentation errors before the subsequent statistical analysis. We calculated the subcortical volumes based on the derived segmentations, known voxel size, and voxel number employing standard functions of *fslmaths* and *fslstats* (FSLUTILS suite).

##### Assessment of subcortical brain iron deposition by susceptibility-weighted imaging

A measure for brain iron depositions was derived from SWI images. SWI images were linearly coregistered to the native space T1 images of the respective subjects using the FMRIB’s Linear Image Registration Tool (Jenkinson and Smith 2001; Jenkinson et al. 2002). For the computation of SWI mean voxel intensities, we used the T1-derived segmentation masks and standard functions of *fslmaths* and *fslstats*. To standardize the voxel intensities of subcortical SWI measures, we expressed the mean voxel intensities as the contrast-to-noise ratio (CNR) referenced to a localized CSF signal. Therefore, we created spheres with a diameter of 4 mm in the MNI space for the lateral ventricles neighboring the subcortical structures of interest (right: x: 93, y: 132, z: 82; left: x: 85, y: 132, z: 82) by *fslmaths* (as highlighted for the axial plane in Fig. 1). We non-linearly normalized the native space T1 images of each subject to the MNI152_T1_1mm template using the FMRIB's Non-Linear Image Registration Tool (FNIRT) suite following the recommendations by Andersson et al. (2007). We transferred the CSF spheres from the standard space to the individual native space for each subject using the *invwarp* function of FNIRT. Correct sphere placement was manually controlled for each subject, and *fslmaths* and *fslstats* standard functions computed the mean voxel intensity and the standard deviation (SD) for the CSF signal. After data extraction, we calculated the CNR for each subcortical ROI following the equation: (mean_ROI_-mean_CSF_)/SD_CSF_. Here, lower CNR values indicate increased iron deposition, and higher CNR values decreased iron deposition.

##### Evaluation of the bioenergetic state by ^31^phosphorus magnetic resonance spectroscopy data

^31^P-MRSI spectra were fitted in the time domain using the well-established AMARES (advanced method for accurate, robust, and efficient spectral fitting) algorithm (Vanhamme et al. 1997) as implemented in the Oxford Spectroscopy Analysis toolbox (Purvis et al. 2017) (OXSA; https://github.com/oxsatoolbox/oxsa) for Matlab^®^. A 1st-order phase correction to compensate for receiver dead-time was performed before spectral fitting. The AMARES fitting algorithm was developed for the evaluation of MRSI spectra. The algorithm allows imposing prior knowledge and boundary conditions on the fitting parameters, i.e. chemical shift, phase, amplitude and line width for each metabolite, to constrain the nonlinear least-squares fit. A Gaussian line-shape for each peak was used. The following metabolites were taken into account: Adenosine triphosphate (ATP), phosphocreatine (PCr), inorganic phosphate (iP), phophocholine (PC), phosphoethanolamine (PE), glycerophosphocholine (GPC), glycerophosphoethanolamine (GPE), diphosphoglycerate (DPG) and nicotinamide adenindinucleotide (NAD). Initial values for the peak positions, i.e., chemical shifts relative to PCr (0.0 ppm), of the phosphorous metabolites were taken from Ren et al. (2015). Due to homonuclear ^31^P-^31^P J-coupling, the α- and γATP signals are split into duplets with the same amplitude, and the βATP signal is split into a 1-2-1 triplet. J-coupling is taken into account and imposed as prior knowledge. The J-coupling constant was constrained to 16 Hz (Jung et al. 1997). All initial values, prior knowledge and boundary conditions are summarized in Additional file 1: Table S1. After fitting the spectra, we determined the area under each signal and calculated metabolite ratios ((βATP + PCr)/iP, βATP/iP, and PCr/iP) for each of the four voxels of interest (VOI) to account for the high degree of within-spectra autocorrelation of metabolites and to standardize the potentially differing alimentary intake of phosphorus-containing nutrients. Based on the intra-individual differing rostral brain length and resulting imprecise localization of distinct subcortical brain structures within the CSI grid, rostrally neighboring (but hemispherically different) VOIs were averaged for subsequent analyses.

### Statistical analysis

We computed all statistical analyses using GraphPad Prism (version 9.0.0 88) on a MacOS Mojave (version 10.14.6) workstation. Demographics and clinical characteristics are reported as mean ± SD. Initially, we analyzed whether hemispherical side differences for distinct subcortical voxels (for ^31^P-MRSI) or neuroanatomical structures (SWI CNR or volumetric measures) were present using paired sample t-tests. As the presence of hemispherical side differences may foster our findings' interpretability, these analyses were exploratory and are subsequently reported as uncorrected p-values.

**Fig. 2:**
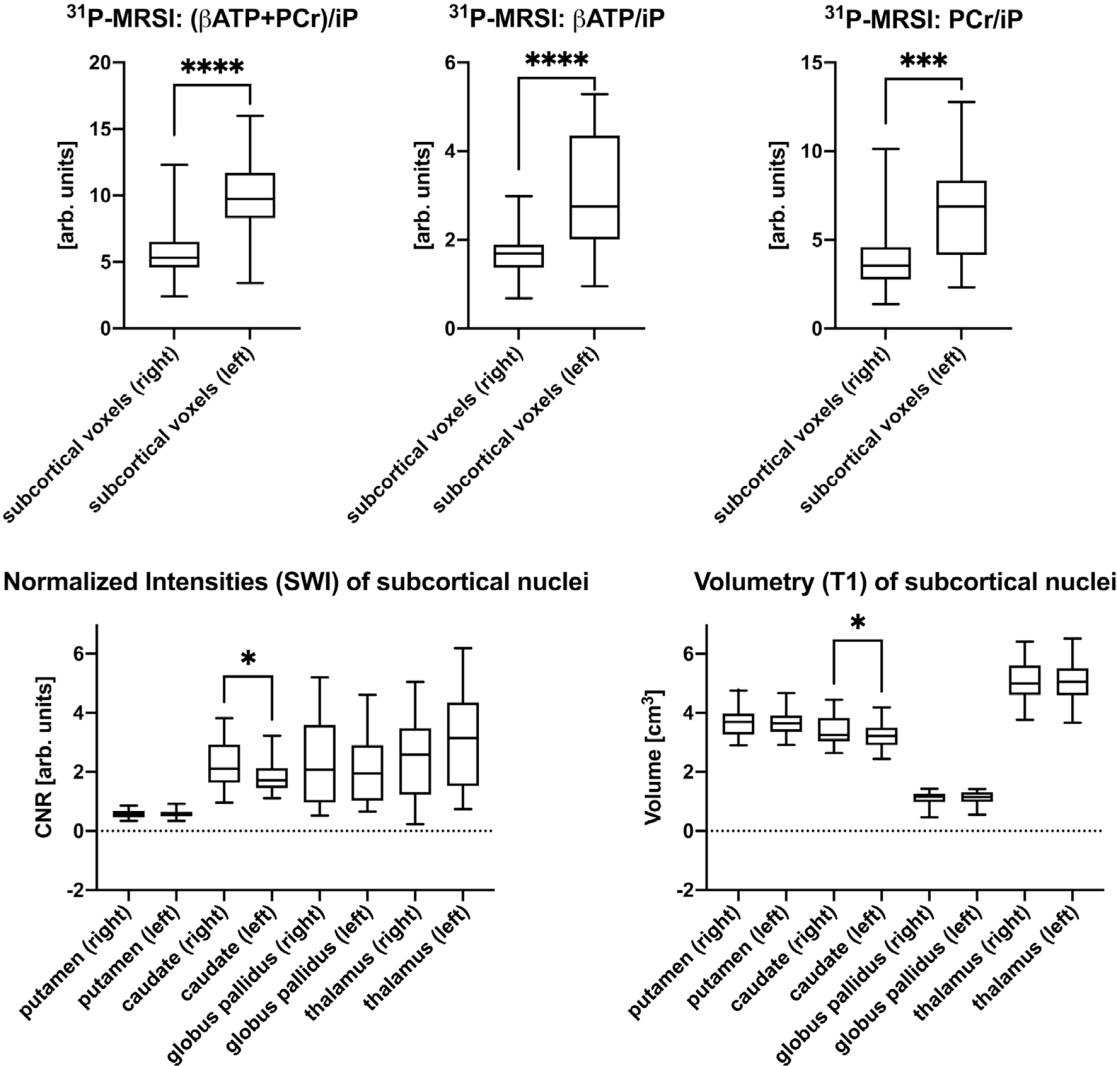
Hemispherical side differences for ^31^P-MRSI measurements, normalized intensities (SWI), and volumetry (T1) of subcortical nuclei. Box plot diagrams are plotted with the median and the 95% confidence interval whiskers. */**/***/***: significance levels (*: P ≤ .05, **: P ≤ .01, ***: P ≤ .001; ****: P ≤ .0001). ^*31*^*P-MRSI*: ^31^Phosphorus magnetic resonance spectroscopy imaging. *arb. units* arbitrary units. *ATP* adenosine triphosphate, *CNR* contrast-to-noise ratio, *iP* inorganic phosphate, *PCr* phosphocreatinine, *SWI* susceptibility-weighted imaging

We computed six multiple linear regression models to test our predefined hypotheses:

The three metabolite ratios served as the dependent variables, the CNR values, or the volumetric measures of the four selected subcortical brain structures served as the independent variables. These results were corrected for multiple comparisons (n = 6) applying the Bonferroni correction, resulting in an adjusted significance level (P_adj_) of P_adj_ ≤ 0.0083. For significant findings on an uncorrected statistical level, we also calculated parameter estimates and the goodness of fit for our multiple regression models to enhance our results' interpretability. In addition, we used a combination of graphical and numerical diagnostics to test the validity of prior assumptions for multiple regression models (absence of multicollinearity, normality of residuals, and the presence of homoscedasticity), which are highlighted in Fig. 2, Additional file 1: Figure S2, Table S2 and Table 1. To explore potential relationships between clinical parameters and PD-related brain changes, we performed correlation analyses for demographic and clinical data with our neuroimaging derived parameters. We performed logistic regression analyses for dichotomous variables (i.e., sex and the more-affected side) and Pearson's correlations for age, disease duration, MDS-UPDRS subscores, Hoehn and Yahr scale, and the LEDD (Schade et al. 2020) with our neuroimaging markers. The exploratory Pearson’s correlation analyses were illustrated via a heatmap (consisting of correlation coefficients). To decrease the number of statistical tests and for the sake of enhanced interpretability, we averaged over both hemispheres.

**Table 1:** Summary of multiple regression model results of (βATP + PCr)/iP vs. SWI CNR values

Analysis of variance	SS	DF	MS	F	p-value	
Regression	48.79	4	12.20	F(4,57) = 41.39	P < .0001****	
SWI: putamen (CNR)	15.46	1	15.46	F(1,57) = 52.46	P < .0001****	
SWI: caudate (CNR)	2.00	1	2.00	F(1,57) = 6.78	P = .0117*	
SWI: globus pallidus (CNR)	2.98	1	2.98	F(1,57) = 10.10	P = .0024**	
SWI: thalamus (CNR)	0.79	1	0.79	F(1,57) = 2.68	P = .1074	
Residual	16.80	57	0.29			
Total	65.58	61				

Parameter Estimates	Variable	Estimate	SE	95% CI	T	p-value
β0	Intercept	1.36	0.31	0.74; 1.98	4.39	P < .0001****
β1	SWI: putamen (CNR)	4.44	0.61	3.21; 5,67	7.24	P < .0001****
β2	SWI: caudate (CNR)	0.27	0.10	0.06; 0.48	2.60	P = .0117*
β3	SWI: globus pallidus (CNR)	0.19	0.06	0.07; 0,31	3.18	P = .0024**
β4	SWI: thalamus (CNR)	0.10	0.05	0.00; 0.18	1.64	P = .1074

Goodness of Fit						
DF	57					
Multiple R	0.86					
*R*^*2*^	0.74					
*R*^*2*^_*adj*_	0.73					
SS	0.74					
RMSE	0.11					

Multicollinearity	Variable	VIF	R^2^ with other variables			
β0	Intercept					
β1	SWI: putamen (CNR)	1.59	0.37			
β2	SWI: caudate (CNR)	1.18	0.15			
β3	SWI: globus pallidus (CNR)	1.29	0.22			
β4	SWI: thalamus (CNR)	1.21	0.17			

Normality of Residuals	Statistics	p-value	Passed normality test (α = .05)?			
Anderson and Darling (1952)	0.58	P = 0.13	Yes			
D’Agostino et al. (1990)	2.23	P = 0.33	Yes			
Shapiro and Wilk (1965)	0.97	P = 0.16	Yes			
Massey (distance) (1951)	0.08	P > .10	Yes			

The table summarizes the multiple regression model of (βATP + PCr)/iP vs. SWI CNR values, including descriptive analyses. Apart from the overall significance test of the model, parameter estimates, goodness of fit, and necessary assumptions for multiple regression models were tested (absence of multicollinearity and normality of residuals). */**/***/***: significance levels (*: P ≤ 0.05, **: P ≤ 0.01, ***: P ≤ 0.001; ****: P ≤ 0.0001)

*CI* confidence interval, *CNR* contrast-to-noise ratio, *DF* degrees of freedom, *MS* mean square, *R*^*2*^ coefficient of determination, *R*^*2*^_*adj*_ adjusted coefficient of determination, *RMSE* root mean square error, *SE* standard error, *SS* sum of squares, *SWI* susceptibility-weighted imaging, *T* t-statistic, *F* F-Statistic, *VIF* variance inflation factor

## Results

Thirty right-handed patients with PD were enrolled of whom 19 (63.3%) were male and eleven (36.7%) female with a mean age of 62.5 ± 9.4 years and a disease duration of 6.9 ± 5.0 years. The disease severity is characterized by MDS-UPDRS-I (7.3 ± 4.1), MDS-UPDRS-II (8.8 ± 6.8), MDS-UPDRS-III (24.5 ± 13.2), MDS-UPDRS-IV (5.1 ± 3.3) scores, and a Hoehn and Yahr Stage of 2.1 ± 0.8. Our study subjects took a levodopa equivalent daily dose (LEDD) of 680 ± 465 mg/d. 57% (n = 17) PD patients presented with left- and 43% (n = 13) with right-lateralized symptoms.

### Hemispherical side differences in subcortical brain bioenergetics, iron deposition, and volumetric measures.

We observed that the ^31^P-MRSI-derived metabolite ratios were significantly lower in the right hemisphere (Fig. 2) inclusive of (βATP + PCr)/iP (t(30) = 5.58, P < 0.0001) (right 5.9 ± 2.6 vs. left 9.9 ± 3.3), βATP/iP and PCr/iP of the (βATP + PCr)/iP ratio with significant side differences for βATP/iP (t(30) = 6.96, P < 0.0001, right: 1.7 ± 0.6, left: 3.1 ± 1.3) and PCr/iP (t(30) = 4.07, P < 0.001, right: 4.1 ± 2.4, left: 6.9 ± 2.8). Representative spectra to illustrate the hemispherical differences are highlighted in Additional file 1: Figure S1. The logistic regressions showed that lateralization of brain energy metabolite levels was not driven by the more severely affected body side of the study participants. The CNR values derived from the SWI images showed similar but less pronounced side differences for the caudate (t(30) = 2.4, P = 0.025, right: 3.4 ± 0.5, left: 3.2 ± 0.5) but not for the other regions of interest. Volumetric measures revealed only significant side differences for the caudate (t(30) = 2.32, P = 0.027, right: 2.2 ± 0.8, left: 1.9 ± 0.6).

### Subcortical brain iron deposition, but not the individual volumetric measurements, predicts the bioenergetic status of each hemisphere

**Fig. 3:**
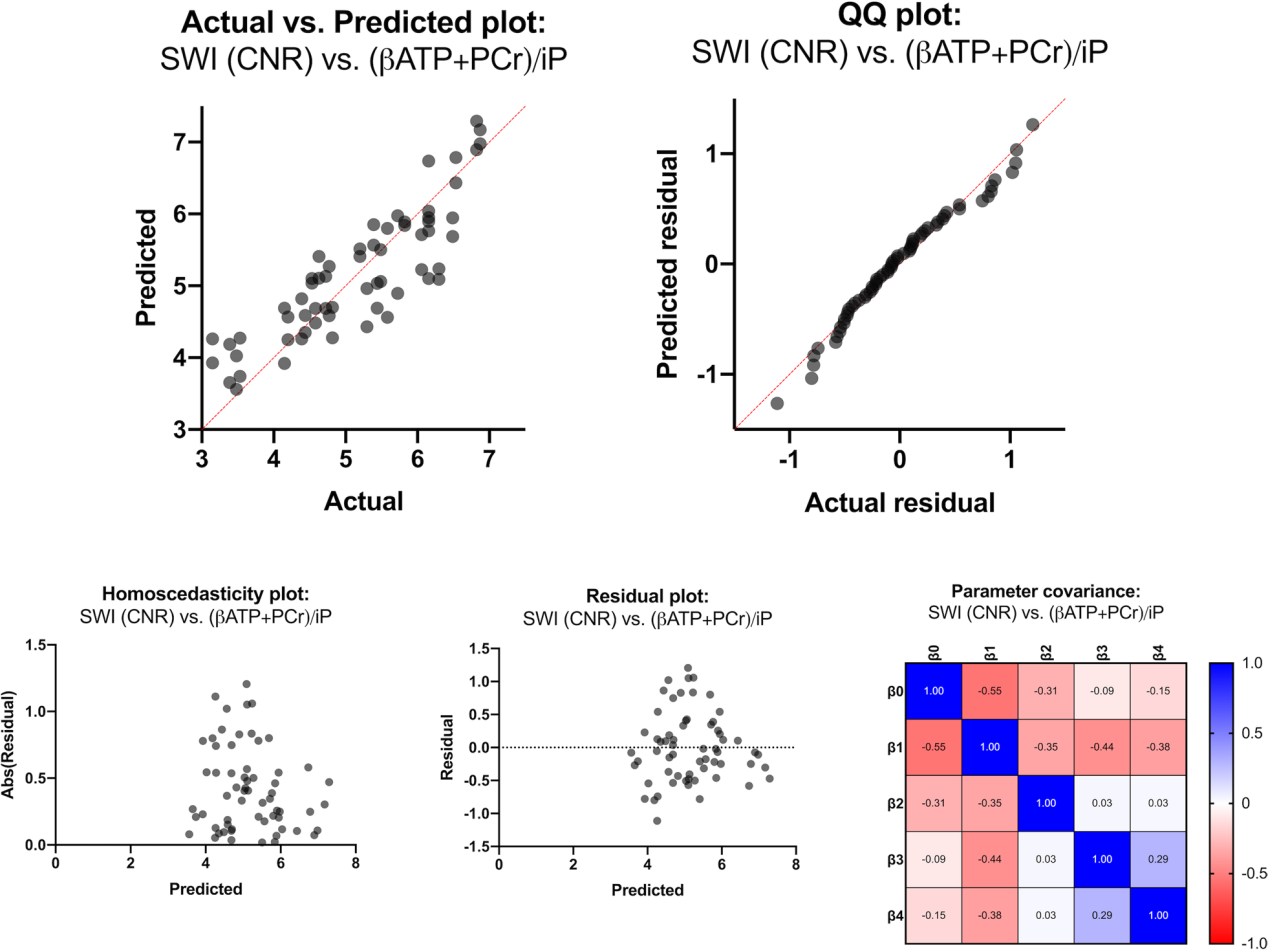
Graphical representation of the multiple linear regression model of (βATP + PCr)/iP vs. SWI CNR values. The validity of the respective multiple regression model is shown in the Actual vs. Predicted plot (the line of identity is highlighted in red). We demonstrated the fulfillment of necessary assumptions for multiple linear regression models by a QQ plot (normality of residuals), a homoscedasticity plot (evenness of residuals' variance), a residual plot (residuals are not themselves predictive), and a parameter covariance matrix (selected parameters are not concerningly intertwined). *Abs(Residual)* absolute value of residuals, *ATP* adenosine triphosphate, *CNR* contrast-to-noise ratio, *iP* inorganic phosphate, *PCr* phosphocreatinine, *SWI* susceptibility-weighted imaging

Based on three metabolite ratios and two independent variable sets, we computed six multiple regression models. We observed a highly significant association for the model of (βATP + PCr)/iP vs. the CNR values (putamen, caudate, globus pallidus, and thalamus) (P < 0.0001) (see Table 1). Here, the parameter estimates for the CNR of the putamen (P < 0.0001), the caudate (P = 0.0117), and globus pallidus (P = 0.0024) significantly contributed to the prediction of our model. The overall goodness of fit resulted in a high adjusted coefficient of determination *R*^*2*^_*adj*_ of 0.74. The independent variables did not show a relevant degree of multicollinearity with variance inflation factors ranging from 1.18 to 1.59 and *R*^*2*^ (among included variables) of only maximal 0.37 (putamen). Diagnostic tests for the normality of residuals were all passed (Table 1, Fig. 3: QQ plot). Furthermore, the present multiple linear regression model showed homoscedasticity (Fig. 3: Homoscedasticity plot), the residuals themselves were not predictive of the dependent variable (Fig. 3: Residual plot), and the selected parameters were not concerningly intertwined (Fig. 3: Parameter covariance matrix). For illustrative purposes, the second (uncorrected) significant (P = 0.0393, *R*^*2*^_*adj*_ = *0.10*) regression model (PCr/iP vs. CNR) is listed in the Supplementary Material (Additional file 1: Table S2, Figure S2). We could describe no significant findings or trends for the βATP/iP ratio vs. CNR regression model and the regression models with volumetric measures as independent variables.

### Neither subcortical brain iron deposition nor the individual volumetric measurements correlate with age, disease duration, or MDS-UPDRS-III

**Fig. 4:**
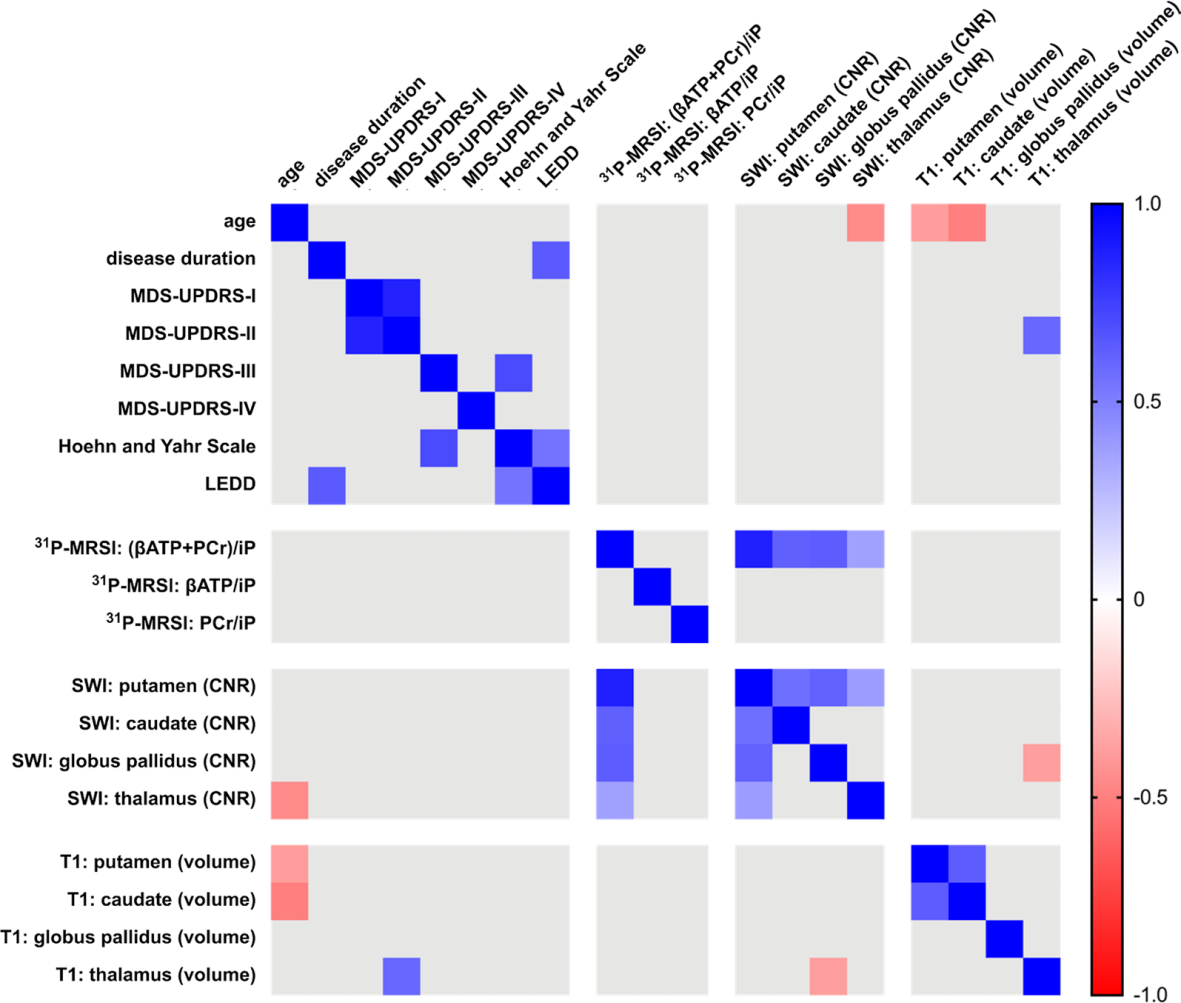
Heatmap for the correlation analyses of demographic, clinical, and neuroimaging data. Presented are Pearson’s correlation coefficients (thresholded with a p-value of > .05) as exploratory analyses (color-coded for negative and positive coefficients, see right scale). ^31^P-MRSI: ^31^Phosphorus magnetic resonance spectroscopy imaging. *CNR* contrast-to-noise ratio, *LEDD* levodopa equivalent daily dosage, *MDS-UPDRS* Movement Disorders Society Unified Parkinson’s Disease Rating Scale, *SWI* susceptibility-weighted imaging

Figure 4 summarizes the exploratory Pearson’s correlations of our neuroimaging measures with demographic and clinical data. The logistic regressions with sex were negative, suggesting that sex did not confound on metabolite ratios and imaging findings. Neither SWI nor T1 imaging findings correlated with demographic or clinical data. Furthermore, we performed additional exploratory correlations due to the lack of a significant relationship with age, disease duration, and MDS-UPDRS-III. Interestingly, the SWI CNR values seemed to be of minor relevance to characterize the disease state with correlation coefficients ranging between ± 0.40. The same could be observed for the ^31^P-MRSI metabolite ratios.

## Discussion

To the best of our knowledge, this study reports first the potential interconnectedness of bioenergetic disturbances and brain iron deposition level in patients with PD using *in vivo* neuroimaging. Here, subcortical brain iron deposition, particularly in the putamen and globus pallidus, was highly predictive of the overall amount of high-energy containing phosphates in our subjects. We observed no association with the individual volumetric measurements, highlighting the potential of ^31^P-MRSI and iron-weighted imaging as pathophysiology-orientated biomarkers. Our findings suggest that brain iron deposition is related to mitochondrial impairment in vivo. However, we could not determine a causal relationship between them. Future studies should address whether these findings might indicate therapeutic advancements to improve mitochondrial bioenergetics in patients by administering chelating agents.

The observed hemispherical differences in brain energy metabolism and iron distribution were unexpected findings. Previous reports suggest that the lateralization of distinct SWI findings is present in patients with PD or Multiple System Atrophy with predominant parkinsonism (MSA-P) (Hwang et al. 2015). In line with our findings, the putaminal tracer uptake of [(123)I]β-carboxymethyoxy-3-β-(4-iodophenyl)tropane PET indicated that the right hemisphere is predominantly affected in PD, being potentially related to handedness (Scherfler et al. 2012). Hemispheric side differences, in particular those concerning the role of the dominant hemisphere, could also be predictive for individual symptom presentation and disease progression (Ham et al. 2015; Riederer and Sian-Hülsmann 2012). Furthermore, the distribution of striatal dopamine content shows an asymmetric distribution in prodromal PD, being relevant for the subsequent motor symptom onset in patients with PD (Haaxma et al. 2010). Nevertheless, it remains elusive whether these previous findings would also result in bioenergetic alterations and should be considered in future multimodal imaging studies. In contrast, the absence of statistically significant correlations of our basal ganglia ^31^P-MRSI neuroimaging findings with the laterality of symptoms implies that dopaminergic dysfunction and bioenergetic depletion are not associated and likely represent independent pathophysiological traits.

However, the observed hemispherical differences yield important implications for future studies: ^31^P-MRSI studies often record a global signal (e.g., by using surface head coils), which might miss lateralized differences concerning an individual's brain anatomy (Rango et al. 2020). Frequent brain iron deposition in midbrain or brainstem structures of diseased individuals (such as in the substantia nigra of patients with PD) might also be a potential limitation, which could be assessed by iron-weighted neuroimaging (Péran et al. 2010; Yoshikawa et al. 2016; Guan et al. 2018). In contrast, ^31^P-MRSI-mediated examination of in vivo bioenergetics is substantially hampered in these brain structures by the relatively low spatial resolution and insufficient tissue homogeneity to yield satisfactory spectral quality for metabolite quantification. Interestingly, our neuroimaging measures were only marginally associated with the phenotype. In particular, the SWI measures are in contrast to previous reports where the iron deposition was related to disease duration or severity (Wang et al. 2016). This finding implicates the need for longitudinal studies that could address whether brain iron deposition is a consequence or rather a primary driver of neurodegeneration. The latter would be especially relevant as patients with increased brain iron deposition in early disease stages could benefit the most from targeted treatment strategies. Given the likely complexity of one individual's disease pathophysiology, it would be crucial to stratify patients by their outweighing etiology to stratify them and subsequently sustain clinical trial success in the future. The temporal dynamics of brain iron deposition and mitochondrial dysfunction are so far unknown. Subcortical iron deposition can be an epiphenomenon or an active driver of bioenergetic failure in patients with PD. The synthesis of Fe-Sulfur clusters is highly energy-dependent, and the depletion of ATP due to mitochondrial dysfunction can lead to an impaired assembly and accumulation of iron in mitochondria (Muñoz et al. 2016). Iron itself can promote the formation of reactive oxygen species, which additionally impacts mitochondrial homeostasis and can cause bioenergetic depletion (Muñoz et al. 2016). Most likely, these processes act as a vicious cycle (Muñoz et al. 2016). To better understand the temporal dynamics of brain iron deposition and mitochondrial dysfunction in the prolonged process of neurodegeneration, longitudinal studies would thus be necessary. Such studies would substantially benefit from the use of quantitative MRI methods, potentially improving the multi-site reliability of the upcoming findings (Barbosa et al. 2015; Weiskopf et al. 2013). As a limitation, SWI is also sensitive to compounds other than iron, e.g. calcium, potentially distorting the local magnetic field and generating image contrast (Liu et al. 2017). The combination of quantitative susceptibility imaging and relaxometry (e.g., by multiparameter mapping) might thus provide more information on the role of brain iron deposition in neurodegenerative disorders (Weiskopf et al. 2013). The combination of different iron-sensitive MRI methodologies might also lead to the specific detection of divalent and trivalent iron atoms in vivo, as preliminarily demonstrated in a phantom MRI study (Dietrich et al. 2017). Especially for the investigation of mitochondrial dysfunction in patients with PD, the role of divalent and trivalent iron in the production of reactive oxygen species might provide more detailed insights into the underlying biology of PD and could be used to map individual treatment responses to oxidative stress-targeted treatment regimes.

## Conclusions

In this study, we demonstrated that subcortical brain iron deposition is highly predictive of mitochondrial impairment in patients with PD in vivo. Our findings highlight the interconnectedness of two important pathophysiological hallmarks of this disorder that were previously implicated by in-vitro and post-mortem experiments. Our preliminary experimental data support the potential use of chelating agents in individualized treatments for patients with PD. However, longitudinal studies are required to address the temporal aspects of the course of the disease and identify the window of opportunity for personalized therapies.

## Supplementary Information

**Additional file 1.** Additional figures and tables.

## Data Availability

The datasets used and/or analyzed during the present study are available from the corresponding author on reasonable request.

## Abbreviations

PD: Parkinson’s disease
SWI: Susceptibility-weighted imaging
LEDD: Levodopa equivalent daily dose
CNR: Contrast-to-noise ratio
FNIRT: FMRIB’s Non-Linear Image Registration Tool
SD: Standard deviation
VOI: Voxel of interest
CSI: Chemical shift imaging
MSA-P: Multiple System Atrophy with predominant parkinsonism
