# Supplementary material for: Relationship between brain iron deposition and mitochondrial dysfunction in idiopathic Parkinson’s disease
Source: Mol Med. 2022 Mar 4;28:28. doi: 10.1186/s10020-021-00426-9 (PMC8895656; doi:10.1186/s10020-021-00426-9)
Supplement: Supplementary file 1 — Additional file 1. Additional figures and tables. [file 10020_2021_426_MOESM1_ESM.docx]

**Additional Tables**

**Additional Table S1.** **Initial values, boundary conditions and prior knowledge imposed for spectral fitting using the AMARES algorithm.** See the table notes for common settings that apply for all metabolites.

|  | **Initial conditions^1)^** | **Boundary conditions^2)^** | **Prior knowledge^3)^** | | |
| --- | --- | --- | --- | --- | --- |
|  | Chemical  shift [ppm] | Chemical  shift range [ppm] | multiplet | Chemical  shift  delta [Hz] | Amplitude ratio |
| βATP | -16.12 | [-18.9,-15.0] | triplet | 15 | 1:2:1 |
| αATP | -7.50 | [-8.5,-7.0] | duplet | 16 | 1:1 |
| γATP | -2.53 | [-3.0,-2.0] | duplet | 15 | 1:1 |
| PCr | 0 | [-1.0,1.0] |  |  |  |
| PC | 6.23 | [5.0,7.0] |  |  |  |
| PE | 6.77 | [5.0,7.0] |  |  |  |
| GPC | 2.94 | [2.5,3.2] |  |  |  |
| GPE | 3.49 | [3.2,4.0] |  |  |  |
| iP | 4.8 | [4.5,5.3] |  |  |  |
| NAD | -8.3 | [-9.0,-8.0] |  |  |  |
| DPG | 5.2 | [5.0,6.0] |  |  |  |

1) Initial values: linewidth 10 Hz, amplitude 10 and phase 0 degrees for all metabolites. 2) Boundary conditions: linewidth [1, 50] Hz, amplitude [1, infinity] and phase [0,360] degrees for all metabolites. 3) Prior knowledge: reference peak PCr and same phase for all metabolites. Abbreviations: Adenosine triphosphate (ATP), phosphocreatine (PCr), inorganic phosphate (iP), phophocholine (PC), phosphoethanolamine (PE), glycerophosphocholine (GPC), glycerophosphoethanolamine (GPE), diphosphoglycerate (DPG) and nicotinamideadenindinukleotid (NAD).

**Additional Table S2. Summary of multiple regression model results** **of PCr/iP vs. SWI CNR values**

| **Analysis of Variance** | **SS** | **DF** | **MS** | **F** | **p-value** |
| --- | --- | --- | --- | --- | --- |
|  |  |  |  |  |  |
| Regression | 84.03 | 4 | 21.01 | F(4,57) = 2.70 | P = .0393* |
| SWI: putamen (CNR) | 51.38 | 1 | 51.38 | F(1,57) = 6.61 | P = .0128* |
| SWI: caudate (CNR) | 30.52 | 1 | 30.52 | F(1,57) = 3.93 | P = .0524 |
| SWI: globus pallidus (CNR) | 35.18 | 1 | 35.18 | F(1,57) = 4.53 | P = .0377* |
| SWI: thalamus (CNR) | 0.00 | 1 | 0.00 | F(1,57) = 0.00 | P = .9924 |
| Residual | 443 | 57 | 7.78 |  |  |
| Total | 527 | 61 |  |  |  |

| **Parameter Estimates** | **Variable** | **Estimate** | **SE** | **95% CI** | **T** | **p-value** |
| --- | --- | --- | --- | --- | --- | --- |
|  |  |  |  |  |  |  |
| β0 | Intercept | 4.47 | 1.59 | 1.28; 7.66 | 2.81 | P = .0069** |
| β1 | SWI: putamen (CNR) | 8.09 | 3.15 | 1.79, 14.39 | 2.57 | P = .0128* |
| β2 | SWI: caudate (CNR) | 1.05 | 0.53 | 0.01; 2.12 | 1.98 | P = .0524 |
| β3 | SWI: globus pallidus (CNR) | 0.65 | 0.31 | 0.04; 1.27 | 2.13 | P = .0377* |
| β4 | SWI: thalamus (CNR) | 0.00 | 0.25 | -0,50; 0,51 | 0.01 | P = .9924 |

| **Goodness of Fit** | |
| --- | --- |
|  |  |
| DF | 57 |
| Multiple R | 0.40 |
| *R^2^* | 0.16 |
| *R^2^_adj_* | 0.10 |
| SS | 443 |
| RMSE | 2.79 |

| **Multicollinearity** | **Variable** | **VIF** | ***R^2^* with other variables** |
| --- | --- | --- | --- |
|  |  |  |  |
| β0 | Intercept |  |  |
| β1 | SWI: putamen (CNR) | 1.59 | 0.37 |
| β2 | SWI: caudate (CNR) | 1.18 | 0.15 |
| β3 | SWI: globus pallidus (CNR) | 1.29 | 0.22 |
| β4 | SWI: thalamus (CNR) | 1.21 | 0.17 |

| **Normality of Residuals** | **Statistics** | **p-value** | **Passed normality test (**𝛂 **= .05)?** |
| --- | --- | --- | --- |
|  |  |  |  |
| Anderson–Darling(42) | 0.68 | P = .07 | Yes |
| D'Agostino–Pearson omnibus(43) | 5.90 | P = .05 | Yes |
| Shapiro–Wilk(44) | 0.95 | P = .02* | No |
| Kolmogorov–Smirnov (distance)(45) | 0.09 | P > .10 | Yes |

The table summarizes the multiple regression model of PCr/iP vs. SWI CNR values, including descriptive analyses. Apart from the overall significance test of the model, parameter estimates, goodness of fit, and necessary assumptions for multiple regression models were tested (absence of multicollinearity and normality of residuals). */**/***/***: significance levels (*: P ≤ 0.05, **: P ≤ 0.01, ***: P ≤ 0.001; ****: P ≤ 0.0001). CI: confidence interval. CNR: contrast-to-noise ratio. DF: degrees of freedom. MS: mean square. *R^2^*: coefficient of determination. *R^2^_adj_*: adjusted coeffcient of determination. RMSE: root mean square error. SE: standard error. SS: sum of squares. SWI: susceptibility-weighted imaging. T: t-statistic. F: f-Statistic. VIF: variance inflation factor.

**Additional Figures**

**
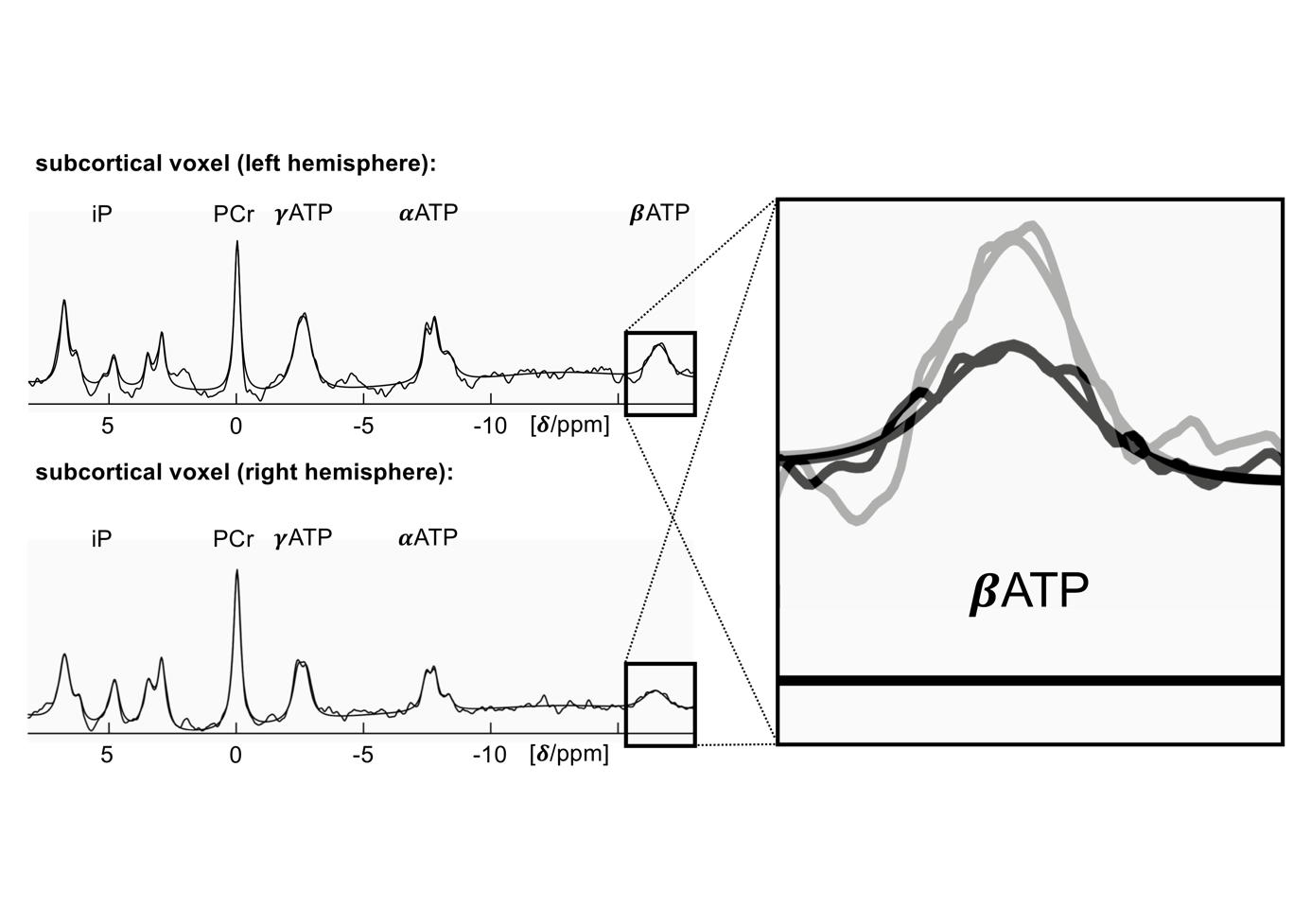
**

**Additional Figure S1. Representative 31P-MRSI spectra of the same subcortical voxel of each hemisphere of a single study participant.** For illustrative purposes, we highlighted the 𝜷ATP peak in a magnified panel and overlaid the respective spectra for each hemisphere. The 𝜷ATP peak of the left hemisphere (light grey) contains a larger area-under-the-curve compared to the right hemisphere (black).

**
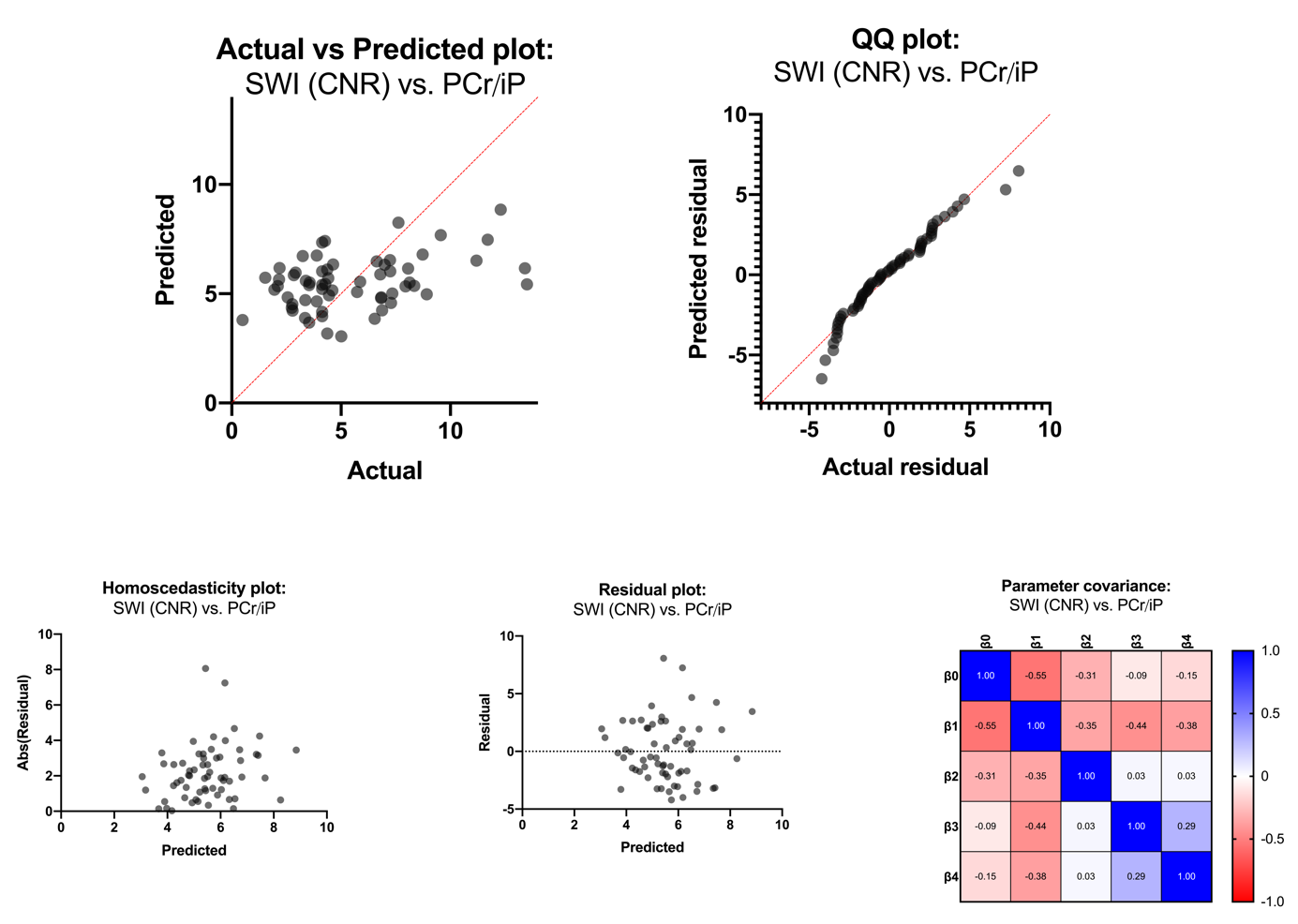
Additional Figure S2. Graphical representation of the multiple linear regression model** **of** **PCr/iP vs. SWI CNR values. The validity of the respective multiple regression model is shown in the actual v. predicted plot (highlighted in red is the line of identity). We demonstrated the fulfillment of necessary assumptions for multiple linear regression models using a QQ plot (normality of residuals), homoscedasticity plot (evenness of residuals' variance), residual plot (residuals are not themselves predictive), and parameter covariance matrix (selected parameters are not concerningly intertwined).** Abs(Residual): absolute value of residuals. CNR: contrast-to-noise ratio. iP: inorganic phosphate. PCr: phosphocreatinine. SWI: susceptibility-weighted imaging.
